# Clinico-epidemiological profile of cerebrovascular accident in eastern Nepal: A descriptive cross-sectional study

**DOI:** 10.1097/MD.0000000000035569

**Published:** 2023-10-13

**Authors:** Vivek K. Rauniyar, Sajjad Ahmed Khan, Sakshyam Gautam, Surya Bahadur Parajuli

**Affiliations:** a Department of Clinical Neurology, Birat Medical College Teaching Hospital, Morang, Nepal; b Birat Medical College Teaching Hospital, Morang, Nepal; c Department of Community Medicine, Birat Medical College Teaching Hospital, Morang, Nepal.

**Keywords:** cerebrovascular accidents, eastern Nepal, epidemiology

## Abstract

cerebrovascular accident (CVA) has contributed to a significant increase in the morbidity and mortality rates in lower middle-income counties like Nepal. Despite being a common noncommunicable disease in Nepal, little attention has been paid to it, in terms of formulating national health plans and policies by the concerned authorities. This was a descriptive cross-sectional study in patients diagnosed with cerebrovascular accidents at a tertiary care hospital in Eastern Nepal. We analyzed 128 diagnosed cases of cerebrovascular accidents from February 26, 2023 to June 26, 2023 after taking ethical clearance from the Institutional Review Committee (Reference no. IRC-PA-283/2078–79). Data were analyzed by Statistical Package for the Social Sciences version 23. The objective of this study was to explore the age and sex distribution of CVA, its association with medical co-morbidities, and known risk factors like Type-2-Diabetes Mellitus, hypertension, thyroid disorders, smoking and alcohol. Together with calculating the distribution of ischemic CVA and hemorrhagic CVA we had also staged the disease based on the National Institute of Health Stroke Scale.

## 1. Introduction

Cerebrovascular accident (CVA) is defined as a rapidly onset clinical sign of focal or global disturbance of cerebral function with no apparent cause other than a vascular origin.^[1]^ As of 2019, there were 12.2 million incident cases of stroke, 101 million prevalent cases of stroke.^[2]^ Based on disability-adjusted life years; stroke is one of the major causes of death and is among the top 5 diseases in Nepal.^[3]^ Over the last few decades, 75% of all stroke deaths and 81% of the total disability-adjusted life years lost due to stroke have occured in developing countries.^[4]^ Inadequacy of knowledge and awareness towards the signs and symptoms of the diseases and ignorance at the early stage are few inevitable factors which hinder the timely diagnosis and management of the diseases.^[5]^ Poor accessibility to health care centers and lack of adequate trained neurologists in the country further add to the problem.^[6]^ There is paucity of data on the CVA clinic-epidemiological profile in eastern Nepal. In this study, we aimed to find out age and sex distribution of CVA, association with medical co-morbidities and known risk factors, types of CVA and severity assessment via the National Institute of Health Stroke Scale (NIHSS). This will raise the concern of community health policy makers and the concerned authorities in addressing the burden of the disease at national level.

## 2. Materials and methods

### 2.1. Study design and population

This descriptive cross-sectional study included patients diagnosed with cerebrovascular accidents at a tertiary care hospital in Eastern Nepal. We included every diagnosed case of CVA via neuroimaging that was treated on an inpatient or outpatient basis. We analyzed 128 diagnosed cerebrovascular accidents cases from February 26, 2023, to June 26, 2023. We included patients with a clinico-radiological diagnosis of CVA presenting to our center. Participants meeting inclusion criteria and were evaluated on-site (outpatient and inpatient departments) using the structured proforma after taking ethical clearance from the Institutional Review Committee (Reference no. IRC-PA-283/2078–79).

### 2.2. Inclusion and exclusion criteria

All the diagnosed cases of cerebrovascular accidents with the clinical features of global or focal neurological deficits, where no any cause other than vascular was identified on radiological evaluation, were included in this study. We excluded the cases presenting with neurological deficit without radiological evidences of vascular insult to the brain.

### 2.3. Data collection

We included all the diagnosed cases of cerebrovascular accidents meeting inclusion criteria, and evaluated them on-site (Outpatient and Inpatient departments) using the structured proforma. Particulars and general information of the patients was assessed to find out age and sex distribution. Associated medical co-morbidities of the patients like hypertension, type-2-diabetes mellitus and thyroid disorders were also recorded. Smoking and alcohol history was also taken to show the association with CVA. Similarly, based on neuroimaging findings, we stratify it into ischemic CVA and hemorrhagic CVA. The NIHSS was used to grade the severity of the stroke as: minor stroke, moderate stroke, moderate to severe stroke and severe stroke.

## 3. Results

The average age of presentation among cerebrovascular accidents cases was 63.35 years and 61.79 years in male and female respectively (Table 1). Out of them, 84 (65.62%) were male and 44 (34.37%) were female (Table 2). The medical co-morbidities; systemic hypertension (68.75%), Type-2-Diabetes Mellitus (39.84%), was prevalent amongst and hypothyroidism (3.13%) were present (Table 3). The known risk factors; intake of alcohol (25.78%) and smoking (32.03%) were found (Table 4). There were 93 (72.65%) and 35 (27.34%) cases of ischemic and hemorrhagic stroke respectively (Table 5). Forty-seven (36.71%) had minor stroke, 60 (46.84) with moderate stroke, 18 (14.06%) with moderate to severe stroke and 3 (2.34%) with severe stroke as per the NIHSS (Table 6).

**Table 1:** Epidemiological characteristics of age distribution among CVA patients (n = 128).

Parameters	Average age of presentation
Male	63.35 yr
Female	61.79 yr

CVA = cerebrovascular accident.

**Table 2:** Epidemiological characteristics of sex distributions among CVA patients (n = 128).

Parameters	n (%)
Male	84 (65.63)
Female	44 (34.37)

CVA = cerebrovascular accident.

**Table 3:** Distribution of known medical co-morbidities among CVA patients (n = 128).

Parameters	n (%)
Systemic hypertension	88 (68.75)
Type-2-diabetes mellitus	51 (39.84)
Hypothyroidism	4 (3.13)

CVA = cerebrovascular accident.

**Table 4:** Distribution of Known risk factors among CVA patients (n = 128).

Parameters	n (%)
Smoking	41 (32.03)
Alcohol	33 (25.78)

CVA = cerebrovascular accident.

**Table 5:** Types of CVA amongst the study population (n = 128).

Parameters	n (%)
Ischemic stroke	93 (72.65)
Hemorrhagic stroke	35 (27.34)

CVA = cerebrovascular accident.

**Table 6:** Assessment of severity of CVA based on the National Institutes of Health Stroke Scale (NIHSS) scoring system (n = 128).

Parameters	n (%)
Minor stroke	47 (36.71)
Moderate stroke	60 (46.87)
Moderate to severe stroke	18 (14.06)
Severe stroke	3 (2.34)

CVA = cerebrovascular accident.

## 4. Discussion

On a global basis, Median age of presentation of CVA is 66 years.^[7]^ Hypertension, cigarette smoking, alcohol consumption, and diabetes are the main predisposing factors for stroke, and ischemic stroke is more common (63%) than hemorrhagic stroke (37%).^[3]^ Talking about the sex predisposition, Women accounted for 57.1% of stroke deaths in 2019, with stroke accounting for 6.2% of all female deaths, while comprising 4.4% of all male deaths. In total, approximately 55,000 more fatal strokes occur in women each year than men.^[8]^ Most (85%) strokes are ischemic, predominantly caused by small vessel arteriolosclerosis, cardioembolism, and large artery athero-thromboembolism while ischemic strokes in younger patients can result from a different spectrum of causes such as extracranial dissection and approximately 15% of strokes worldwide are the result of intracerebral hemorrhage, which can be deep (basal ganglia, brainstem), cerebellar or lobar.^[9]^ Stroke prevention is all about avoiding modifiable risk factors but management of stroke depends on treating the pathophysiology which is not very well understood.^[10]^ Reperfusion remained the mainstay of treatment; however the efficiency of thrombolytic drugs depends on many factors including the age of the clot, the specificity of the thrombolytic agent for fibrin and the presence and half-life of neutralizing antibodies.^[11]^ The most effective thrombolytic drug recommended by the by the US National Institute of Neurological Disorders and Stroke is recombinant tissue plasminogen activator like alteplase.^[12]^ Unlike, fibrin activators like alteplase, reteplase and tenecteplase which convert plasminogen to plasmin directly, non-fibrin activators like the drugs streptokinase do so indirectly and are relatively less effacious.^[11]^ Apart from the conventional reperfusion techniques, embryonic stem cells, mesenchymal cells and induced pluripotent stem cells has assessed their potential for tissue regeneration, maintenance, migration and proliferation, rewiring of neural circuitry and physical and behavioral rejuvenation.^[13]^

Noncommunicable diseases were once regarded to be the diseases of western world but with the change in our lifestyle and dietary measures, these disorders have found their way to low-income countries like Nepal and Cerebrovascular accidents are no exception. Because of sedentary lifestyle and westernization of diets, hypertension, Diabetes and hyperlipidemia are on the rising secular trend which has led to subsequent increased incidence of thromboembolic events. Clinical pictures of CVA are very nonspecific, to begin with, like mild weakness of arms, and are often ignored at an early stage of the disease because of lack of awareness regarding the clinical presentation of the diseases, so these patients always come to us at a very advanced stage. Not only that, lack of accessibility to health care centers in the periphery of the country, limited number of neurologists in the countries and poor patient compliance towards treatment and follow-up are few other inevitable factors which make the management approach even more challenging. Despite such a high burden of the diseases, little attention has been given to noncommunicable diseases in the context of Nepal and our health plans and policies are very much biased towards infectious diseases. Further, the epidemiology of Cerebrovascular accident, age of presentation, sex predisposition as well as relation with comorbid conditions in low to middle-income countries such as Nepal have not been closely analyzed. In this study we have analyzed the socio-demographic aspects and medical co-morbidities associated with cerebrovascular accidents. The clinco-epidemiological picture is similar to international standards, only difference being we found male predominace over female, in terms of sex predisposition. With the evidence-based data presented in this study, it will draw the attention of concerned authorities towards CVA and will help community health policy-maker in addressing the burden of cerebrovascular accidents.

## 5. Conclusion

Average age of presentation of cerebrovascular accidents in male was slightly lower than that of females. In our settings, males are more likely to have the disease compared to females. Talking about the association with medical co-morbidities, systemic hypertension, Type-2-Diabetes Mellitus and hypothyroidism were the common co-morbidities associated with CVA. Similarly, smoking and alcohol history were also present in many of the sample population. Ischemic stroke was more common than hemorrhagic stroke and major bulk of patients had minor and moderate stroke as per the NIHSS. Appropriate screening techniques in high-risk populations, early diagnosis and treatment of the diseases can lead to significant decrease in national burden of the diseases and subsequent drop in morbidity and mortality due to the diseases.

## Acknowledgments

The Authors are grateful to the department of Radiology for providing the needful information.

## Author contributions

**Conceptualization:** Sajjad Ahmed Khan.

**Data curation:** Vivek K Rauniyar, Sakshyam Gautam.

**Formal analysis:** Sakshyam Gautam.

**Investigation:** Vivek K Rauniyar, Sakshyam Gautam, Surya Bahadur Parajuli.

**Methodology:** Vivek K Rauniyar, Surya Bahadur Parajuli.

**Writing – original draft:** Vivek K Rauniyar, Sajjad Ahmed Khan.

**Writing – review & editing:** Vivek K Rauniyar, Surya Bahadur Parajuli.
